# Supplementary material for: Molecular detection and genetic characterisation of a large flood-borne outbreak of human leptospirosis in Jakarta, Indonesia: A retrospective analysis of surveillance data
Source: PLoS Negl Trop Dis. 2026 May 13;20(5):e0014243. doi: 10.1371/journal.pntd.0014243 (PMC13170961; doi:10.1371/journal.pntd.0014243)
Supplement: Attachment — Submitted filename: Response to reviewer.docx [file pntd.0014243.s008.docx]

Dear Dr. Elsio Wunder Jr,

We thank the editorial team and the external reviewers for the opportunity to revise our manuscript entitled “**Molecular detection and genetic characterisation of a large flood-borne outbreak of human leptospirosis in Jakarta, Indonesia: a retrospective analysis of surveillance data**”. We appreciate the thorough evaluation and constructive feedback provided by the editors and reviewers.

We have carefully considered all comments and have made substantial revisions to address the points raised.

Below, we provide a detailed point-by-point response, highlighting the changes implemented and clarifying aspects where necessary.

Thank you for your consideration of the revised manuscript (R1).

Your sincerely,

On behalf of all authors,

Suwarti

**Additional Editor Comments (if provided)**

**Editor comment #1**

In addition to the reviewer's comments, please clarify:
A description of the laboratory diagnostic effort during the period will provide more context for interpretation. Is it consistent across all districts? do all clinically suspect patients get lab tested with IgM and PCR or is it up to the clinician to request? Indicate the nature of the reporting to the MoH. Mandatory for all lepto categories?

**Author response #1**

We have included a brief description of the laboratory diagnostic approach as part of the leptospirosis surveillance in the Methods section (lines 112-116). Reporting to MOH of leptospirosis cases is mandatory. According to the MOH surveillance guidance, all patients with suspected leptospirosis will undergo diagnostic testing, which was consistent across all districts. In reality, some patients were not tested, mostly because of logistical reasons such as stockouts of kits or reagents. We added this last point to the study limitations (line 295-297).

**Editor comment #2**

Figure 3 is confusing. The boxes from PCR, MLST are coming from Probable. It should show first boxes for the various tests and end with the case classifications.

**Author response #2:**

We have made the suggested modifications to Figure 3.

**Editor comment #3**

Probable cases: It seems most were IgM+ but the breakdown for the other 2 possible criteria should be clear because they are technically still clinically suspect only.

**Author response #3**:

We have clearly defined the criteria for probable cases in accordance with MoH guidelines as illustrated in Figure 3. Specifically, 91.5% (140/153) of cases were classified as probable based on positive IgM test results and 8.5% (13/153) based on clinical symptoms.

**Editor comment #4**

Table 1: Methods include MAT but Table 1 does not show MAT results.

**Author response #4**:

We confirm that MAT was not conducted as part of the testing approach on the current outbreak as stated in the Methods section (line 126).

**Editor comment #5**

Figure 5: The information is just a few numbers and they are already provided in the text. It appears that the figure is not necessary.

**Author response #5**:

As suggested, we removed the Venn diagram (former Figure 5) from the manuscript.

**Methods**

**Reviewer 2 comment #1:** The objectives were clearly described. The study design was appropriate. The statistical analysis was correct. And there is no concerns about ethical issue. However, the concatenated MLST sequence alignment and phylogenetic analysis were not described thoroughly for example R library used or software (which command) used. And these command and library should be cited properly.

**Author Responses #1:**

As requested by Reviewer 2, we have revised the description of the data analysis in the Methods section (line 167-174). We provided additional details on the concatenated MLST sequence alignment and the MLST scheme. Furthermore, we included information on the R package used, specifying the relevant command libraries.

**Results**

**Reviewer 1 comment #1**

I have requested additional analysis and modification of the figures 1.

**Author Response #1**:

We have modified the Figure 1 as suggested by the reviewer.

**Reviewer 2 comments #2**

The results were still not analyse thoroughly.

There was no demonstration on the precipitation in sub-distrist to demonstrate the relation between (suspected, probable and confirmed) cases and precipitation, or between cases and flooded areas. If author can present in a series of weekly accumulate case numbers for each subdistrict, it will show how the outbreak occurred.

**Author Response #2**

We have added further details to the Results (lines 178-185), as follows. The outbreak reached its peak between January 10 and 14, 2020 (week 3-4), about two weeks after the first heavy flooding event (week 2). Subsequent floods occurred in the second half of January, 2020 (week 5-7), although with fewer subdistricts affected and fewer cases. More floods occurred in February (week 8-10), affecting a significant number of subdistricts but with few new cases. We also added Supplementary Figure S1 to illustrate the outbreak pattern, showing the series of weekly accumulated cases in relation to flooded areas and precipitation.

**Reviewer 2 comments #3**

I suggest to show the border line of each five districts on the Jakarta Map to be clear for readers

**Author response #3**

As suggested, we have added the border line between the five districts to Figure 2.

**Reviewer 2 comments #4**

In Table 1: the Fever onset section, three groups (<4, 4-7, >7) might give clearer picture for concerning performance of RDT and PCR in each group case.

**Author response #4**

We appreciate the reviewer’s suggestion. We included an extra Table (Table 2) that shows a breakdown of test performance by duration of fever <7 vs ≥7 days (there were no cases who had less than 4 days of fever).

**Reviewer 2 comments #5**

Table 1 lacking outcome after treatment and the final diagnosis. This will be useful for investigate the performance of RDT as it could give a high false positive in some brand.
**Author response #5**

Unfortunately, the available data on the outbreak did not include clinical outcomes of cases (eg disease severity and death). This means we were not able to perform further analyses on RDT performance in relation to clinical outcomes.

**Reviewer 2 comments #6**

In Figure 4, it is better to use the different color among the suspected, probable, and confirm groups in comparison.

**Author response #6**:

We have modified Figure 4 as suggested.

**Reviewer 2 comments #7**

Authors can identify the Leptospira species from the amplified MLST loci and report in the Table 2 and further analysis. For five MLST samples, two separate tree should be reconstructed based on 5 and loci, respectively.

**Author response #7**

We have modified the separate phylogenetic tree based on the 5 MLST (currently Table 3) and loci in Figure 5.

**Conclusions**

**Reviewer #1 comment #1**

I have requested to add some comments about the potential use of these techniques for future studies.

**Author response #1**

We have added a statement on the potential use of RT-PCR and RDT for leptospirosis diagnosis, emphasizing that the combined use of both methods are critical for improving case detection during outbreaks (line 281-282).

**Reviewer #2 comment #2**

It might be much better to see the more discussion on how the increase cases occured only after the first heavy rain (1 Jan 20) but not the later heavy rains (and after several floods). Add more discussion on the RDT and PCR accuracy related to the fever onset

**Author response #2**:

We have elaborated on the observation that there was an early peak in cases after the first heavy rainfall and flooding, followed by an extended outbreak with less precipitation and lower case numbers (line 257-260). This biphasic pattern is likely associated with overflowing of sewage systems during heavy rainfall, as reported in a previous study. Additionally, we discussed the use of RDT and RT-PCR in relation to fever onset. Our findings are consistent with previous studies, showing that the use of RT-PCR improved case detection and confirmation by 3-6% (line 278).

**Summary and General Comments**
**Reviewer #1 comment #1**

Specific comments:

1) Lines 40-42. Improve the sentence for clarity

**Author response #1**

We made edits to improve clarity (lines 42-44).

**Reviewer #1 comment #2**

2) Lines 276-277. Discuss about the delay in leptospirosis cases after the rain, for example it seem unlikely that people are getting infected with rain water that washes off urine from soul.

**Author response #2**

We have added the delay in the detection of leptospirosis cases of approximately 10-14 days between heavy flood events and the peak. This is likely due to the delayed recognition that an outbreak had started, alongside the incubation period of the infection (line 260-265).

**Reviewer #1 comment #3**

Figure 6. Include information about date (months and years) and location, for sample sequences, in the phylogenetic tree. You could use branch color. Please discuss if there is any pattern.

**Author response #3**

We have included months, years, location and branch color in the phylogenetic tree. The phylogenetic analysis indicates that *L. borgpetersenii* emerged during the initial outbreak in the Central and North Jakarta subdistricts around mid-January 2020 (week 4), whereas *L. interrogans* was transmitted in the West and Central Jakarta subdistricts during the outbreak occurring from mid- to late January 2020 (week 6) (lines 240-242).

**Reviewer #1 comment #4**

Create phylogenetic trees with sequences from samples that produced less that 3 alleles in PCR, to visualize the diversity of leptospiral strains (include dates in these trees) and place them as supplementary figures

**Author response #4**

We created the phylogenetic trees of the samples that produced less than 3 alleles in PCR (Figure S2).

**Reviewer #1 comment #5**

Table 2. Please explain the logic for serovar determination (species determination many times is not correlated with serologic classification)

**Author response #5**

We have changed Table 2 to Table 3 to accommodate the Reviewer #2’s comment #4 in Results section. The serovar determination were based on sequence type (ST) matching in the Leptospira PubMLST database as described in line 247.

**Reviewer #1 comment #6**

Discuss if there is clinical differences between L. interrogans and L. borgpetersenii infections.

**Author response #6**

We did not observe any clinical symptoms differences between *L.interrogans* and *L. borgpeterstenii* infections, although the numbers were too low to draw any firm conclusions.

**Reviewer #1 comment #7**

Please discuss further if you recommend the use of serology IgM combined with RT-PCR to detect leptospirosis cases.
**Author response #7**

We have added a statement on the potential use of RT-PCR and RDT for leptospirosis diagnosis, emphasizing that the combined use of both methods are critical for improving case detection during outbreaks (line 281-282).
