# Supplementary material for: Molecular detection and genetic characterisation of a large flood-borne outbreak of human leptospirosis in Jakarta, Indonesia: A retrospective analysis of surveillance data
Source: PLoS Negl Trop Dis. 2026 May 13;20(5):e0014243. doi: 10.1371/journal.pntd.0014243 (PMC13170961; doi:10.1371/journal.pntd.0014243)
Supplement: Attachment — Submitted filename: Response to reviewer APR26.docx [file pntd.0014243.s009.docx]

Dear Dr. Elsio Wunder Jr,

We thank the editorial team and the external reviewers for the opportunity to revise our manuscript entitled “**Molecular detection and genetic characterisation of a large flood-borne outbreak of human leptospirosis in Jakarta, Indonesia: a retrospective analysis of surveillance data**” (PNTD-D-25-01675R1). We appreciate the thorough evaluation and constructive feedback provided by the editors and reviewers.

We have carefully considered all comments and have made substantial revisions to address the points raised.

Below, we provide a detailed point-by-point response, highlighting the changes implemented and clarifying aspects where necessary.

Thank you for your consideration of the revised manuscript (R3).

Your sincerely,

On behalf of all authors,

Suwarti

**Reviewer comments**

**Methods**

**Reviewer 1 comment #1:**

It was not clear to me whether they submitted the DNA sequences to GenBank or any other public database.

**Author response #1:**

Since the complete allele assignment was not achieved, resulting in an incomplete MLST scheme 3 profile, the allele sequences could not be submitted to a PubMLST database, as described in lines 178-180.

**Reviewer #2 comment #2:**

Please add a proper citation for R and R library used in the manuscript.

**Author response #2:**

We have added the citation (line 180-183), as follows: The phylogenetic tree was constructed in R version 4.4.3 (R Core Team, Vienna, Austria) [23] using Biostrings version 2.74.1 [24] to manage the sequence data, phangorn version 2.12.1 [25] for phylogenetic tree construction, and ape version 5.8.1 [26] for tree visualisation.

**Reviewer #2 comment #3:**

Redraft the author summary as it is currently as a short version of abstract.

**Author response #3:**

We have revised the author summary as described in lines 58-71 of the manuscript.

**Results**

**Reviewer #1:** no comment

**Reviewer #2 comment #1**: Table 1 -Should be clear in the last section that are the result of 95 samples, which be used for both PCR and RDT.

**Author response #1:**

We have now clearly stated in Table 1 that 95 samples were tested with both PCR and RDT

**Reviewer #2 comment #2**: Table 2 -Please re-check the result of PCR as the total should be 29 positive PCR

**Author Responses #2:**

We confirm that we obtained a positive PCR result for 29 samples.

**Reviewer #2 comment #3:** Please remove every row of "Any fever onset"

**Author response #3:** As suggested, we have removed this from Table 2.

**Reviewer #2 comment #4: As there were <4 days of fever onset, I suggest group as i) 4-7 days fever onset and ii) >7 days fever onset**

**Author response #4:** We have modified the groups as suggested in Table 2.

**Conclusions**

**Reviewer #1 comment #1**

In the discussion section it is necessary to address the evidence of different leptospiral strains causing infection in Jakarta form December 2019-February 2020. Please indicate whether other studies, in Indonesia or other parts of the world, have detected different strains affecting the same community at the same time. Also indicated the possible explanations for the diversity of leptospiral strains.

**Author response #1:** We have elaborated on possible explanations for the different *Leptospira* strains in lines 303-305 and 312-316.

**Reviewer #1 comment #2**

In discussion it will be interesting to know whether one of the leptospiral species caused more severe disease.

**Author response #2:** We agree with the reviewer. However, the limited sample size did not allow us to explore the association between disease severity and the leptospiral species. We included this in the study limitations (line 318-319)

**Reviewer #1 comment #3:**

Fig S2 , The authors must indicate the genes used to build the tree. This figure shows that sequences from Jakarta fall in 3 clusters, 2 of them are identified as *L. interrogans*, the other one as L. borgpetersenii. The upper cluster remains unidentified, even though it is very conspicuous. I recommend to run Blast some of these sequences to see whether they belong to another leptospiral species.

**Author response #3:**

We have added BLAST analysis of the *secY* gene as the most consistently amplified gene in the remaining samples beyond those identified by MLST. We modified this in the Method section (line 175-178) and our BLAST results confirm that the upper clusters of the Fig S2 correspond to *Leptospira interrogans* (line 251-255).

**Summary and general comments**

**Reviewer #2 comment #1** After heavy rain and flooding, upserged suspected, probable and confirmed leptospirosis cases were reported. However, 11.3% were confirmed cases (32/282 positive PCR), while 49.6% were probable cases (140/282 positive RDT). It is common that RDT is not sensitive if the fever of onset less than 3-5 days. And it is common for false positive in the endemic area, tropical countries. The balance pro and cons of each assay use should be discuss and suggestion. When we should use PCR or RDT (single or pair sera) for giving more accurate early diagnosis & treatment or disease surveilance. What is the key message or lesson learn from this manuscript?

**Author Responses #1:**

We have added the pros and cons of RDT and RT-PCR use for early diagnosis and surveillance programs in the discussion section, lines 286-302. The key message from this study is to combine the use of both tests; RT-PCR to improve diagnostic sensitivity mainly in early symptom onset and serological diagnostic tools for case detection during outbreaks.

**Reviewer #2 comment #2** To demonstrate "a large flood borne" outbreak, the incidence of leptospirosis in West Jakarta are the highest in this study. However, authors did not compare to the incidence in the same duration in different years to point out the sharp increasing of cases (suspected, probable or confirmed cases). Is topographic map can explain why the leptospirosis case number higher in the west than the other area.

**Author response #2:** We have added the discussion of the topographic map of West Jakarta that facilitate the severe flood compare to other districts due to the slow water drainage, and presence of great lakes and rivers as observed in previous study. This enabled *Leptospira* to spread in the regions as also supported by possible of more active surveillance in the district. We modified the discussion in line 270-273.

**Reviewer #2 comment #3**
It is interesting that more than one species of genus Leptospira identified. Author should more describe or discuss (or speculate) on relatedness of ST identified in the regions. For examples, the two close related isolate (55 and 71) of ST 149 found in west and central Jarkata, While two close related isolate of ST 193 found in north and central Jarkata. Interestingly, the central area, where the close related ST 149 and 193 identified, had few area effected by flooding.

**Author Responses #3:** We thank the reviewer for this insightful comment and agree that the distribution of sequence types (STs) across districts is an interesting epidemiological observation. However, we believe that our current dataset does not allow robust interpretation of the geographic relatedness of the identified STs.

Possible explanations include high mobility of the patient population obscuring the spatial relationship between the detected strains (ST) and the transmission location (we did not collect detailed individual-level exposure histories), as well as the possibility of continuous circulation of multiple strains among the huge rat populations residing in the city.
